# Developments of reproductive management and biotechnology in the pig

**DOI:** 10.21451/1984-3143-AR2019-0055

**Published:** 2019-10-23

**Authors:** Olli Peltoniemi, Stefan Björkman, Marianne Oropeza-Moe, Claudio Oliviero

**Affiliations:** 1 Department Production Animal Medicine, Faculty of Veterinary Medicine, University of Helsinki, Finland.; 2 Norwegian University of Life Sciences in Sandnes, Norway.

**Keywords:** large litter, sow, piglet, management, biotechnology

## Abstract

This review aims to describe changes in production environment, management tools and technology to alleviate problems seen with the present hyperprolific sow model. Successful parturition in the pig includes the possibility to express adequate maternal behaviour, rapid expulsion of piglets, complete expulsion of placenta, elimination of uterine contamination and debris, neonatal activity and colostrum intake. We focus on management of large litters, including maternal behaviour, ease of parturition, colostrum production, piglet quality parameters and intermittent suckling. There are also some interesting developments in technology to assess colostrum and immune state of the piglet. These developments may be utilized to improve the success rate of reproductive management around farrowing, lactation and after weaning. We also discuss new insights in how to examine the health of the mammary gland, uterus and ovaries of hyperprolific sows. Finally, we assess the latest developments on breeding and technology of hyperprolific sows, including artificial insemination (AI), real-time ultrasound of the genital tract and embryo transfer (ET). We conclude that 1) for the sow to produce sufficient colostrum, both the behavioural and physiological needs of the sow need to be met before and after parturition. Furthermore, 2) new ultrasound and biopsy technology can be effectively applied for accurate diagnosis of inflammatory processes of the udder and uterus and timing of AI regarding ovulation to improve insemination efficiency. Finally, 3) developments in cryopreservation of germ cells and embryos appear promising but lack of valid oocyte collection techniques and nonsurgical ET techniques are a bottleneck to commercial ET. These latest developments in management of parturition and reproductive technology are necessary to cope with the increasing challenges associated with very large litter sizes.

## Introduction

The pig appears to be superior in its reproductive ability at least when compared to other domestic animal species. This ability is based on the extremely high rate of fertility. Over the past three decades, efficient breeding and management has almost doubled the litter size of the domestic European sow breeds (Oliviero, 2019). During the same period, the duration of farrowing (second stage, from the first to the last fetus expulsed) has extended remarkably and is now four to five times longer than in the early 1990s (Oliviero *et al*., 2019). This may have resulted in an increase in farrowing complications such as postpartum dysgalactia syndrome (PDS, Kaiser *et al*., 2018a, b) and retention of placenta and a decrease in subsequent fertility (Björkman *et al*., 2017c; 2018c). Along with this development, we have seen a constant downward trend in the birth weight of the piglets and a similar trend in colostrum intake, which are connected and are the most important risk factors for piglet mortality (Oliviero *et al*., 2019). In the other hand, we have seen a tremendous increase in efficiency of production, which has considerably improved farming economy and related industry in a highly positive way. However, this may have come, at least to some extent, at the expense of animal health and welfare.

A large litter may be challenging for the metabolism of the sow such that there may be difficulties in resumption of ovarian cyclicity after weaning, especially in young sows in certain European breeds (Oliviero *et al*., 2013; Peltoniemi *et al*., 2016; Björkman *et al*., 2018c; Oliviero *et al*., 2019). Therefore, there appear to be major challenges associated with increasing litter sizes that are evident at farrowing, lactation and after weaning, which are periods when the foundations of the subsequent pregnancy are laid (Algers and Uvnäs-Moberg, 2007; Martineau *et al*., 2012). This paper discusses some of the key applications of reproductive biotechnology for the modern hyperprolific sow and her numerous offspring (beyond 17 piglets in a “large litter”). The first focus is on management and technology-related innovations used to address the challenges that sows and piglets face in terms of the ambient parturition environment and development of immunity around parturition and lactation. These innovations include optimizing colostrum intake and evaluation of colostrum yield and quality produced by the sow. Among the newer management interventions, intermittent suckling is aimed at not only hastening the production cycle, but also more importantly to improve the resilience of piglets after weaning. Secondly, we review some novel approaches to examine ovarian, uterine and mammary gland function *in vivo*. These involve both sampling and diagnostic imaging techniques that have been recently either discovered or considerably developed. Finally, we provide an updateon the use of artificial insemination (AI), which has been successful regarding use of fresh semen since inception of this technique, and future prospects of embryo transfer (ET) in the pig.

## Management of large litters

### Developments in parturition management of hyperprolific sows

Prolonged farrowing increases the risks of piglet asphyxia during parturition and less vital piglets at birth (Herpin *et al*., 2001). Yun et al. (2014) demonstrated that providing space and abundant nest building material before farrowing tended to increase sow plasma oxytocin concentrations (25 *vs*. 18 pg/ml in sows with abundant nesting material *vs*. sows with crates, respectively). Abundant nesting material also increased piglet serum IgG and IgM concentrations during early lactation (15 *vs*. 10 mg / ml (IgG) and 0,9 *vs*. 0,7 mg/ml (IgM) of sows with abundant nesting material vs. with sows in crates, respectively; Yun *et al*., 2014). Allowing for the intrinsic nesting behaviour to occur can reduce farrowing duration and therefore allow for more vital piglets (Jensen, 1986; Islas-Fabila *et al*., 2018) and for greater colostrum intake due to a shorter time interval gap from the start of farrowing to first suckling (Manjarin *et al*., 2018). Uncomplicated farrowing also reduces pain and inflammation in the sow (Björkman *et al*., 2017c; Kaiser *et al*., 2018a). Allowing the sow to farrow free and providing a substrate (straw, sawdust, paper) 1 to 2 days before farrowing can support the physiological nest building behaviour of the sow. This can significantly reduce farrowing duration and stillbirth rate (Oliviero *et al*., 2008; Gu *et al*., 2011).

With increasing occurrence of large litters, providing the sow with a good basis to produce enough colostrum is fundamental. Loss of back fat in late gestation and consequently sows arriving at farrowing with inadequate body condition affect colostrum yield (Decaluwé *et al*., 2013). Therefore, it appears essential that sows improve their body condition gradually during the whole pregnancy, arriving to farrowing in good body condition (backfat of 17 ± 3 mm) to fulfil protein turnover and sufficient colostrum yield (Oliviero *et al*., 2010; Decaluwé *et al*., 2013). During late pregnancy, not only adequate energy intake but also feeding composition seems to be of key importance in supporting the physiology of farrowing and colostrum quality. Many studies reported that specific essential fatty acids (conjugated linolenic, pinolenic and oleic acids) supplemented in gestating and lactating diets can improve sow colostrum immunoglobulins, piglet performance, average daily gain and weaning weight (Bontempo *et al*., 2004; Corino *et al*., 2009; Yao *et al*., 2012; Hasan *et al*., 2018). The feeding timing during pregnancy and especially in relation to parturition also seems to be of relevance regarding the success of farrowing. Feyera et al. (2018) observed that if the time lapse between the last feeding occasion prior to onset of farrowing lapsed beyond 3 hours, there was a positive linear correlation for time lapse and farrowing duration (Feyera *et al*., 2018). Glucose metabolism was considered to be of highest relevance behind this finding. However, other factors such as feeding fibre (involving bacterial metabolism of the GI tract) were also suggested to support more successful, quicker process of farrowing (Feyera *et al*., 2018). In conclusion, a proper ambient environment regarding food, metabolism, enrichment and space around farrowing are of key importance for successful processes of farrowing and colostrum yield, intake and quality.

### Improving colostrum intake

Increased competition for colostrum intake is a critical factor for neonate piglets. These piglets are born without the protection of maternal immunoglobulins, as the epitheliochorial nature of the porcine placenta does not permit transfer of such large molecular weight structures from maternal to foetal blood circulation. Neonate piglets must acquire maternal immunoglobulins from ingested colostrum for passive immune protection before they produce sufficient endogenous immunoglobulins at approximately 3 to 4 weeks of age (Rooke and Bland 2002; Oliviero, 2013). The concentration of IgG piglet plasma shortly after birth is positively correlated with survival. Dead piglets have lower serum IgG concentrations than their surviving fellow piglets, which indicates low colostrum intake (Vallet *et al*., 2013). At farrowing, the IgG levels in colostrum are approximately 60 to 80 mg/ml. Within 10 to 12 h later IgG levels are reduced by half (35 to 40 mg/ml) and after 24 h a 70% reduction occurs (10 to 16 mg/ml), which is no longer an adequate level (Devillers *et al*., 2011; Quesnel *et al*., 2011; Hasan *et al*., 2016). Therefore, in large litters with prolonged farrowing of more than 6 hours, the immunity and viability of piglets are compromised. Furthermore, hyperprolific sows give birth to more piglets with low birth weight and with signs of intrauterine growth restriction (IUGR). There is an inverse relationship between number of piglets born in a litter and piglet birth weight; large litters are also associated with high variation in piglet birth weight within the litter (Quesnel *et al*., 2008; Akdag *et al*., 2009; Beaulieu *et al*., 2010; Smit *et al*., 2013; Matheson *et al*., 2018). A greater number of piglets born than the available teats at the sow’s udder, lower birth weight and greater birth weight variation increase piglet competition for colostrum intake (Declerck *et al*., 2017). Similarly, lower birth weight and long farrowing duration are associated with lower piglet viability at birth, which can delay the access to the udder (Hoy *et al*., 1994; Islas-Fabila *et al*., 2018). Therefore, all underprivileged piglets should be provided with additional support to acquire a sufficient amount of good quality colostrum (e.g., should be assisted in suckling). To provide the best passive immunity, the procedure of split and assisted suckling should be effectively operated within the first 6 hours from the beginning of parturition, when the colostrum immunoglobulin content is at the maximum (Devillers *et al*., 2011; Quesnel *et al*., 2011; Hasan *et al*., 2016). As small piglets or those with IUGR have difficulties to suckle from large nipples, the smallest functioning nipples should be used when assisting suckling. In conclusion, due to decreasing birth weight and colostrum intake per piglet, colostrum management around farrowing is of key importance for survival of piglets.

### Technology to assess colostrum quality and immune state of neonatal piglets

Both colostrum yield and IgG content vary greatly among sows (Foisnet *et al*., 2010). Factors that affect the total colostrum yield are attributed to environment-related factors as well as to sow and piglet characteristics (Devillers *et al*., 2007; Farmer and Quesnel, 2009; Quesnel, 2011). IgG concentration in maternal colostrum significantly affects the acquisition of passive immunity (Kielland *et al.,* 2015) and therefore knowledge on IgG content of colostrum may be essential to determine the correct action to reduce piglet pre-weaning mortality. The major practical point in assessing colostrum IgG content at the farm level may be identifying the sows with low colostrum IgG levels. Those sows are a risk for a successful acquisition of passive immunity in the piglets. This is of great importance particularly when large litters are present and cross-fostering and split suckling are common management practices employed to maximize colostrum intake. Therefore, if the estimated colostrum IgG content appears to be insufficient, a farmer with this advance knowledge can pay additional attention to the relevant management practices. Hasan et al. (2016) have proposed the use of a Brix refractometer to estimate IgG content in sow colostrum. When used in non-sucrose-containing liquids, the Brix percentage approximates the total solids (TS) percentage (Quigley *et al*., 2013; Hasan *et al*., 2016). At the start of farrowing, immunoglobulins represent a significant portion of the TS (Klobasa *et al*., 1987) and IgG represents 80% of the immunoglobulins in sow colostrum (Porter, 1969; Curtis, 1970). Colostrum samples evaluated with a Brix refractometer are positively correlated with the IgG level measured with ELISA (Hasan *et al*., 2016). Therefore, the Brix refractometer can be an inexpensive, rapid and satisfactorily accurate method for estimating IgG concentration. Differentiation between good and poor IgG content of colostrum is possible by interpreting the results with the categories proposed in Table 1. Hasan et al. (2016) proposed this classification following the nature of the IgG physiological curve during the first 24 h post-partum, when IgG levels peak in the first 3 h and decrease rapidly until values of 10 mg/ml are reached 24 h post-partum (Quesnel *et al*., 2015; Hurley, 2015). Brix values of <20% were correlated with very low IgG levels (14.5 mg/ml), which are not expected during early colostrogenesis. In conclusion, the Brix refractometer is an acceptable method to assess colostrum IgG content at the herd level during the initial hours of parturition, when IgG levels are expected to peak.

**Table 1:** Brix value categories to estimate sow colostrum IgG content according to Hasan et al. (2016). This interpretation scale is valid if the sample is obtained within 0-3 hours from the start of farrowing using a Brix refractometer with a scale range 0-53% (adapted from Hasan *et al*., 2016).

Brix %	IgG estimation categories
< 20	Poor
20-24	Borderline^a^
25-29	Adequate
≥ 30	Very good

a The category “Borderline” should not always be considered to estimate a not adequate IgG content, especially if the found Brix values are on the highest range of this category (23-24%), on the contrary levels falling at the lowest range of this category (20-21%) can be considered not optimal. Taking another sample, after 1-2 h, can allow better interpretation of the results, to see if the development of the estimated IgG content is stable, increasing or decreasing from the initial value (Hasan *et al*., 2016). In conclusion, IgG can be considered as a reliable indicator of colostrum quality. Use of Brix refractometers provide an effective tool to assess colostrum quality, which is essential in the management of a large litter.

### Intermittent suckling

Management strategies to support large litters are numerous. They include at least use of nurse sows (Schmitt *et al*., 2019a, b), split suckling (Donovan and Dritz, 2000), use of substitute milk and automated milk replacers (Difilippo *et al*., 2015) and general neonatal management (Kirkden *et al*., 2013). Among the strategies, intermittent suckling (Kemp and Soede, 2012) is especially interesting, since it may provide a useful tool to postpone weaning of piglets, which becomes relevant for the industry based on the decreasing trend in colostrum intake and birth weights of piglets (Oliviero *et al*., 2019). Therefore, applying an intermittent suckling (IS) protocol, which encourages sows to become pregnant in the middle of lactation, seems like an appealing alternative.

However, IS also involves resumption of reproductive function in the middle of lactation, which may become a further metabolic challenge for the sow. Alternative reproductive management strategies as IS have a considerable impact on grouping dynamics and reproductive functions in the pig (Peltoniemi *et al*., 2016). Sows are in anoestrus during lactation and maturation of follicles is bound to the process of weaning. It is only after weaning that follicles are provided with circumstances for growth and ovulation. This process heralding ovulation stems mainly from the continuous lack of suckling stimulus on the udder, high intake of feed rich in energy and daily application of boar stimulus.

Ovulation in the middle of lactation can be induced by essentially the same means as used after weaning, specifically temporary, transient interruption of suckling stimulus, high feed intake and proper application of boar stimulus. Recent studies (see Kemp and Soede 2012 for a review) have demonstrated that intermittent suckling can induce lactation oestrus especially when IS starts around the normal weaning and is combined with adequate boar stimulation. Oestrus may be induced in up to 90% of the older sows (Gerritsen *et al*., 2008; Soede *et al*., 2012) and over 70% in first parity sows (Chen *et al*., 2017) within 6 days during lactation; farrowing rates and litter size are comparable to controls. Thus, success is dependent on parity as primiparous sows do not appear to respond as well as older sows and there seems to be differences in the response to the IS protocol and in the breed used. The success rate of IS also seems to depend on the management issues around IS (van Nieuwamerongen *et al*., 2014). These include a proper audio-visual isolation of sow and the piglets during IS. Furthermore, group management during boar stimulation around separation time is essential for IS success (Tabe 2; Hasan *et al*., 2019; van Nieuwamerongen *et al*., 2014). In conclusion, lactation oestrus has the potential advantage that the lactation period can be extended while sows are pregnant and this allows piglets to be more developed when eventually weaned. Piglets seem to respond well in terms of growth performance and resilience to the opportunity for extended, although interrupted, suckling possibilities (van Nieuwamerongen *et al*., 2014).

**Table 2:** Descriptive result of individual herd data for a successful intermittent suckling program. Data presented in mean ± SD. Data adapted from Hasan *et al*., 2019.

	Herd number					
Type of production	1	2	3	4	5	6
	Traditional	Traditional	Traditional	Traditional	Traditional	Intermittent suckling
Gestation length, days	115	115.6	116.2 ± 0.1	115	114.4 ± 0.1	115.2 ± 0.2
Farrowing duration, min	211.9 ± 10.7	200.6 ± 12.9	329.2 ± 24.2	261.7 ± 22.1	306.7 ± 27.4	287.8 ± 23.9
Litter size	16.1 ± 0.5	16.7 ± 0.6	14.6 ± 0.6	17.1 ± 0.6	16.5 ± 0.5	16.1 ± 0.5
Live-born piglets	15.3 ± 0.5	15.5 ± 0.5	13.1 ± 0.5	16.5 ± 0.6	14.9 ± 0.4	15.4 ± 0.5
Stillborn piglets	0.8 ± 0.1	1.1 ± 0.2	1.4 ± 0.2	0.6 ± 0.2	2.7 ± 0.5	0.6 ± 0.2
Birth interval, min	14.4 ± 0.9	13.7 ± 0.8	26.4 ± 2.7	16.6 ± 1.8	18.6 ± 1.2	-
Birth time, min	112.1 ± 3.1	100.3 ± 2.9	180.8 ± 7.9	142.2 ± 6.7	147.5 ± 4.1	-
Litter characteristics						
Piglet BW_B_ (live born), g	1445.7 ± 14.1	1275.0 ± 12.4	1413.6 ± 14.5	1220.48 ± 16.5	1279.2 ± 10.4	1446.1 ± 21.7
Piglet weight (weaning: ear tagged), g	6918.8 ± 105.8	6757.4 ± 106.3	7718.4 ± 161.2	5392.0 ± 149.2	7939.5 ± 55.28	6061.0 ± 135.5
ADG^*^ (ear tagged), g	257.8 ± 4.3	246.1 ± 4.6	212.9 ± 5.0	224.0 ± 7.5	228.2 ± 1.7	246.3 ± 7.1
Piglet age (weaning) days	21.0 ± 0.03	21.6 ± 0.02	29.6 ± 0.09	18.1 ± 0.09	28.9 ± 0.03	19.4 ± 0.2
CY^**^, g	4658.5 ± 221.5	4009.4 ± 145.9	4132.2 ± 223.1	4336. 1 ± 268.4	4710.6 ± 129.4	3846.5 ± 367.3
CI^***^, g	332.0 ± 6.6	274. 3 ± 5.8	343.5 ± 7.2	270.9 ± 8.1	331.1 ± 4.5	262.5 ± 10.0
						

ADG* average daily gain, CY** colostrum yield, CI*** colostrum intake.

## Management of hyperprolific sows after parturition

### Mammary gland function

The most important disease of the mammary gland of the postpartum sow is generally considered to be mastitis as part of PDS (Farmer *et al*., 2019), although the role of mastitis as part of the complex in modern sow lines has recently been questioned (Kaiser *et al*., 2018a,b). This disease (PDS) is suggested to be associated with large litters as a connection between farrowing duration and PDS has been established (Tummaruk *et al*., 2013; Björkman *et al*., 2018c). Diagnosis of mastitis is based mainly on clinical signs, as has been reviewed by Gerjets and Kemper (2009).

**Figure 1:**
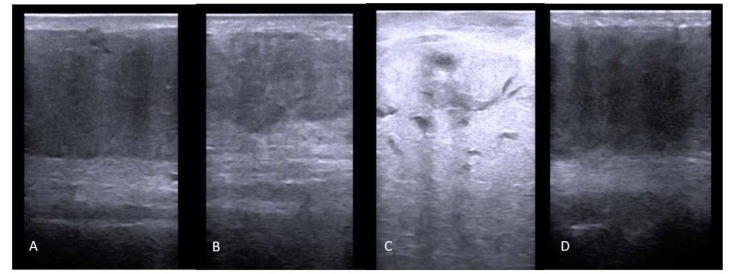
Ultrasound images of a mammary gland of a healthy sow (A) and from sows with from severe udder oedema (B-C). These images show thickened dermal and subdermal tissues (B, C), hyperechoic lobuloalveolar tissue (B, C) with enlarged blood vessels (C) and severe shadowing (D) (Björkman *et al*., 2017a, 2018a).

Recently, other methods such as ultrasound examination and biopsy isolation have been tested for feasibility as diagnostic tools for udder diseases (Baer and Bilkei, 2005; Spiegel *et al*., 2017; Björkman *et al*., 2017a, 2018a, 2018b). In the study by Baer and Bilkei (2005), the sows that had PDS had more hyperechoic images in the ultrasonographic examination of their mammary glands than sows without PDS. Björkman et al. (2017a) made the same observation in sows suffering from severe udder oedema prior to parturition, which is considered a risk factor for subsequent mastitis. In this case report, ultrasound of the mammary glands showed thickened dermal and subdermal tissues, hyperechoic lobuloalveolar tissue with enlarged blood vessels and severe shadowing (Fig. 1). Sows with severe udder oedema also had lower colostrum quality (Björkman *et al*., 2018a). Therefore, PDS must be prevented to ensure the immunity of newborn piglets.

The objective of the study by Spiegel et al. (2017) was to verify by comparative bacteriological examinations of milk samples and mammary gland biopsies whether a better assessment of bacteriological status is possible using biopsies. Diagnostic investigations based on bacteriological examination are complicated, as a similar bacterial content can be detected in milk samples from both healthy and diseased sows. Contamination during sample collection may be a possible reason. Spiegel et al. (2017) obtained biopsies after local anaesthesia using a 7-cm biopsy needle and revealed that biopsy samples of the mammary gland did not provide advantages for bacteriological diagnosis compared to milk sampling. Furthermore, Spiegel et al. (2017) observed complications such as abscess formation following biopsy. The same method was also tested by Björkman et al. (2018b) using an automatic needle with a 14-gauge diameter, 10-cm length and a 22-mm penetration depth. Biopsies were obtained from the lateral-caudal part of three different mammary glands. Before biopsy, glands were disinfected three times with a povidone-iodine solution but no local anaesthesia was used. Sows were monitored until weaning and no complications (such as abscess formation) were observed. There was also no effect of the biopsy before parturition on colostrum production (Han *et al*., 2018). Biopsies can thus be collected in a rapid and humane way. This method seems to be of minor value for diagnosis of mastitis but can be used to study mammary gland function for research purposes, especially for comparison of sows with low and high colostrum or milk yield. Ultrasound imaging of the mammary glands can provide an effective tool for diagnosis of inflammatory processes of the udder, such as PDS.

### Uterine function

In recent years progress has been made in the use of ultrasonography to examine the non-gravid uterus. Timely and correct diagnosis of uterine disease, especially post-partum uterine disease, is essential to prevent subsequent subfertility (Kauffold and Wehrend, 2014). Oliviero et al. (2013) have shown that prolonged parturition can reduce subsequent fertility in the sow that may be associated with an increased incidence of post-partum uterine disease (Björkman *et al*., 2018c). In addition to prolonged parturition, obstetrical intervention, retained placenta and ≥2 stillborn piglets at birth have been shown to affect the incidence of post-partum endometritis (Björkman *et al*., 2018c). Ultrasonography is considered the best tool for diagnosis, not only for endometritis but also for cases in which placenta is retained (Björkman *et al*., 2017c). Examination of uterine structures currently utilizes the following three criteria: fluid echogenicity, echotexture and size (Kauffold and Althouse, 2007). Changes in echotexture reflect changes in endometrial oedema. Increased echotexture, unless attributed to circulating oestrogens originating from enlarged follicles, must be considered abnormal (Kauffold and Althouse, 2007). Furthermore, any fluid echogenicity, unless attributed to pregnancy, semen or oestrus, must be considered abnormal and indicative of an exudative inflammation of an acute or acute-chronic type (Kauffold and Althouse, 2007). Fluid echogenicity is often associated with uterine oedema and therefore increased echotexture and size of uterine cross-sections (Björkman *et al*., 2018c). In contrast, chronic endometritis, representing the most common type of uterine inflammation in pigs, cannot be definitively diagnosed by ultrasonography based on any of the criteria described above (Kauffold and Althouse, 2007). Therefore, it is essential to recognize acute endometritis in time. This can be achieved based on the criteria mentioned above. However, fluid echogenicity, uterine oedema and increased uterine size during the first few days after parturition are not unusual or abnormal (Björkman *et al*., 2018c). Furthermore, when interpreting uterine size, the age and parity of the sow and the number of postpartum days must be considered. Björkman et al. (2018c) provide some reference values for the first postpartum week in Large White x Yorkshire sows.

Recently, the feasibility of transabdominal Doppler sonography (colour, power, pulse wave) to define uterine perfusion characteristics throughout the oestrous cycle in gilts (German Landrace x Pietrain) has been tested (Herlt, *et al*., 2018). These characteristics were perfused area, blood-flow velocity and intensity and resistance and pulsatility index. Colour Doppler sonography was the only feasible technique, as it was less affected by animal movements than power and pulse wave sonography. As determined by colour Doppler sonography, all five parameters determined showed specific patterns throughout the oestrous cycle. Perfused area and blood-flow velocity and intensity increased in proestrus, decreased in oestrus and remained low in midoestrus and most parts of dioestrus. The resistance and pulsatility index showed inversely paralleled patterns. Herlt et al. (2018) encourage the use of colour Doppler sonography for studying uterine capacity or uterus-related infertility, such as in cases of clinically unapparent endometritis. In conclusion, real-time ultrasound examination of the uterus is a fast, practical, efficient and accurate tool for diagnosis of acute inflammatory processes after parturition. Further developments in ultrasound technology, such as use of colour Doppler, may broaden the use of this technique beyond diagnosis of clinical disease of the uterus. In the future, it would be desirable to develop a uterine biopsy method for the sow for diagnosis of chronic uterine disease, like in the equine (Rua *et al*., 2018).

### Ovarian function

Ovarian function postpartum can be monitored using ultrasonography. The use of B-mode ultrasound to determine follicular and corpus luteum size and the factors that affect the size of these structures have been reviewed (Soede *et al*., 2011; Langendijk, 2015; Soede and Kemp, 2015).

Recently, transabdominal colour Doppler sonography was used to assess ovarian blood flow characteristics during the course of the oestrus cycle in gilts (Stark, *et al*., 2019). These characteristics were perfused area, blood-flow velocity and intensity and resistance and pulsatility index. All parameters showed oestrous cycle-dependent patterns. Perfused area and blood-flow velocity were highest in diestrus, followed by proestrus, whereas the patterns of resistance and pulsatilty index were inversely proportional. Stark et al. (2019) concluded that ovarian blood flow was dependent on the stage of the oestrous cycle and was highest during the luteal phase and thus encouraged the use of colour Doppler ultrasonography to also investigate the reasons for ovary-based infertility, including corpus luteum insufficiency or seasonal effects on ovarian function.

**Figure 2:**
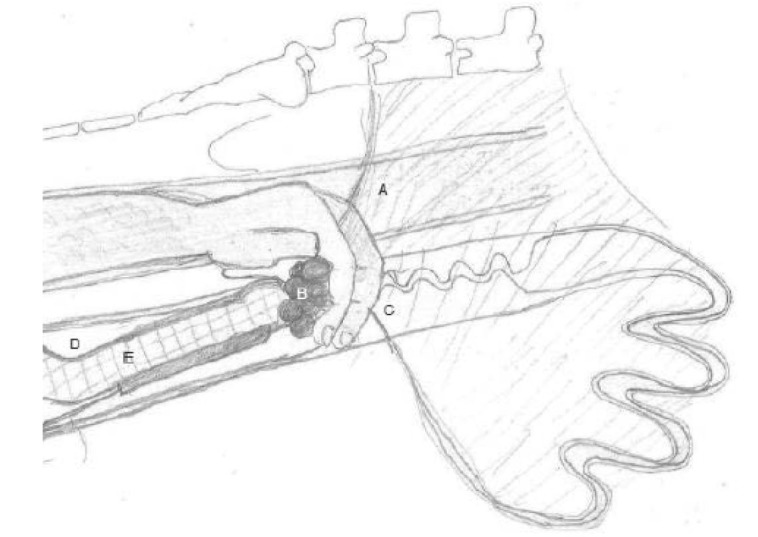
Illustration of the positioning of the transvaginal ultrasound-guided biopsy device. One hand is placed in the rectum (a) and the suspensory ligament of the ovary is palpated. After pulling on the ligament, the ovary is located and the proper ligament of the ovary held between the index and middle fingers, with the ovary on the palm side of the hand. The ovary (b) is pulled along the uterine cervix (c) into the peritoneal part of the pelvic cavity towards the cranial part of the vagina (d). With the other hand, the probe (e) is inserted into the vagina adjacent to the caudal part of the uterine cervix until the ovary becomes visible on the ultrasound screen (Björkman *et al*., 2017b).

Another technique that has recently been used is transvaginal ultrasound-guided biopsy of ovarian tissue. Björkman et al. (2017b) developed this method to obtain luteal tissue and to study corpus luteum function (Fig. 2). Biopsies were performed in four multiparous sows on days 9 and 15 of three consecutive oestrous cycles and the size and histological composition of the samples obtained were evaluated and the reproductive tract of the sows was monitored. Furthermore, biopsies were performed on 26 multiparous sows on days 10 and 13 after insemination and pregnancy rate, gestation length and subsequent litter size were evaluated. Altogether, tissue samples were obtained in 50% of the biopsy attempts. Sows from which one or more samples were obtained were older, heavier and had higher back fat compared to sows where no samples were obtained. No effects of the biopsies were observed on the cyclicity or reproductive organs of the sows or on subsequent corpus luteum diameter, pregnancy rate, gestation length and subsequent litter. The samples obtained had a diameter of 1 mm and contained heterogeneous tissue with various cell types. Björkman et al. (2017b) concluded that a transvaginal ultrasound-guided biopsy method for ovarian tissue can be used to study ovarian function. This method is relatively fast, minimally invasive and humane (Yun *et al*., 2017). Nevertheless, it should be noted that this method may not be used in young and small animals and tissue may be obtained in only half of the attempts. Furthermore, methods to select cells (e.g., laser microdissection) may be used to separate luteal from other ovarian cell types. In conclusion, advanced ultrasound techniques such as colour Doppler may be used to study ovarian dysfunction and seasonal infertility. A transvaginal ultrasound-guided biopsy of ovarian tissue has been developed for the pig and can be used for research purposes.

## Breeding hyperprolific sows

### Artificial insemination

The pig is considered an intrauterine ejaculator (Senger, 2012). Therefore, deposition of semen in the uterus may be considered more of a physiological method than using the caudal portion of the cervix as the primary site of semen deposition. Generally speaking, intrauterine insemination (post-cervical, semen deposited into the uterine base) and deep intra-uterine (semen deposited into uterine horn) have been practiced to allow for a reduction of sperm number per dose, improved fertility or both (Watson and Behan, 2002; Martinez *et al*., 2002; Peltoniemi *et al*., 2009). A similar technique was developed to allow transcervical ET (Martinez *et al*., 2004). Results by Watson and Behan (2002) suggested that 2 or 3 billion spermatozoa/dose using intrauterine AI improve live-born litter size when compared with 1 billion spermatozoa/dose. However, it was subsequently shown that the number of spermatozoa may be reduced to 500 million spermatozoa/dose without detrimental effects on fertility (Martinez *et al*., 2006; Sumransap *et al*., 2007; Tummaruk *et al*., 2007; Roca *et al*., 2016; García-Vázquez *et al*., 2019). Post-cervical insemination seems to provide a number of advantages, such as a reduced sperm number requirement, less time required to perform insemination and faster genetic improvement (reviewed by García-Vázquez *et al*., 2019).

AI is used widely and globally by the industry. Despite these developments, some constraints such as cryopreservation of porcine semen prohibit efficient use of AI in international trade. Current research is focused on issues that affect AI such as freezing rates, cryoprotectants and storage (Yeste *et al*., 2016). Addition of antioxidants and the role of seminal plasma are being explored. As in other species like the horse, there seems to be large individual variation in semen freezability between boars (Yeste *et al*., 2016).

Timing of AI is another important factor in ensuring good fertility. Inseminating too early may not be successful, whilst if the sow is bred too late after ovulation, endometritis resulting in decreased litter size may be observed. Currently, two inseminations per oestrus is a commonly used practice to achieve a high pregnancy rate and large litter size. In a typical sow in oestrus, standing oestrus lasts for about 48 hours on average and ovulation occurs when two thirds of the standing oestrus has passed (Peltoniemi and Kemp, 2019). However, variation in the weaning to oestrus interval may affect the timing of AI. The later the sow enters oestrus after weaning, the sooner the optimal window for insemination (Roca *et al*., 2016). Ultrasound technology, in addition to a fixed-time AI after hormonal treatment protocol, may be used to pinpoint the optimal timing for AI in a specific herd, allowing for a good outcome after a single AI/oestrus (De Rensis and Kirkwood, 2016; Peltoniemi and Kemp, 2019). In conclusion, despite its wide use, application of AI in terms of dose deposition site within the uterus, cryopreservation of spermatozoa and timing towards a single AI are being further developed to advance the use of AI technology.

### Use of ultrasound in the boar

Due to the increase in AI use in the pig breeding industry, there is interest in identifying males with suboptimal fertility to discard them or reduce their use (Pinho *et al*., 2018). This is especially important if a low number of spermatozoa per insemination dose is used and to meet the genetic potential of hyperprolific sows. In addition to proper mating management and insemination technique, high-quality semen from genetically superior sires is of high importance. Assessment of semen quality is one of the major evaluations for the selection of boars for breeding. For this reason, methods to assess the quality of semen before a boar starts reproductive life, or before using his semen for AI are required to predict their “fertility potential” (Pinho *et al*., 2018).

Therefore, studies in the past have focused on examining pre-pubertal and pubertal boars with ultrasound (Clark and Althouse, 2002; Clark *et al*., 2003; Ford and Wise, 2011; Kauffold *et al*., 2011; Pinho *et al*., 2018). The aims of these studies were to establish normal ultrasound parameters to identify subfertile boars and to establish correlations between these parameters and subsequent semen parameters. The first ultrasonographic evaluation of normal boar testes was performed more than 30 years ago. Cartee et al. (1986) compared the ultrasonographic appearance and testicle measurements with semen parameters in 14 Landrace boars but did not find any correlations. Nevertheless, they found significant differences in these parameters between 9-month-old and 15-month-old boars. Likewise, Clark et al. (2003) found an increased paired-testicular diameter in 18-month-old boars compared to 12-month-old boars. However, no correlation between paired-testicular diameter and the average total sperm number was established (Clark *et al*., 2003). Ford and Wise (2011) assessed pubertal development of boars derived from ultrasonographic determination of testicular diameter and length in 160 boars at 4, 5, 6, or 7 months of age. Boars were subsequently castrated and the weight of the testes, mean diameter of seminiferous tubules and percentage of the testis occupied by tubules were determined. At 4 and 5 months of age, although testicular diameter correlated positively with diameter of seminiferous tubules, this relationship was not significant at older ages.

Previously, Kauffold et al. (2011) conducted a study to describe the echogenicity pattern of the epididymis in boars using B-mode ultrasound together with grey-scale analysis. Ejaculate parameters were also determined for investigating the relationships between them and ultrasonographic findings. In the ultrasound images, all parts of the epididymis appeared homogeneous and regular in echotexture. However, while the echotexture of the caput and the corpus was normal, the cauda had a rather marbled echotexture (Kauffold *et al*., 2011). The echogenicity, expressed as the mean grey value, was different in comparison between the three segments of the epididymis (caput > corpus > cauda). The echotexture of the caput of the epididymis correlated slightly positively with the ejaculate volume and the total sperm count. Thus, ultrasound examination of the epididymis with analysis of caput echotexture provides information on semen parameters before semen collection.

Ultrasound examination of the accessory sex gland has also been successfully conducted and the appearance of each accessory sex gland has been described (Clark and Althouse, 2002) but no correlations with semen parameters have been made. It is unlikely that ultrasound examination of accessory sex glands can be implemented into practice. This method is quite challenging and dangerous as it requires rectalizing the boar. It is also not applicable in pubertal boars because of the anatomically small pelvic canal. This method may only be used in adult boars as a diagnostic tool in the work-up of subfertility. In conclusion, ultrasonographic determination of testicular diameter can be used to monitor testicular development during puberty but no correlations have been established with total sperm number in the ejaculate or with subsequent reproductive performance. It would be of particular interest to study whether ultrasound of the epididymis could also be used in prepubertal or pubertal boars to predict their future “fertility potential.”

### Embryo transfer in sows

Global need for foods and animals requires the development of strategies beyond traditional breeding to ensure offspring of high genetic quality and productivity while preserving genetic diversity. Demand for pork has been rising in recent decades due to changes in consumption patterns as incomes increase in developing countries with rapidly growing economies. Genetics from superior sows best meeting with breeding goals are sought internationally. The export of live animals is contentious due to animal welfare issues, biosecurity, economy and sustainability due to long transport times and crossing of borders. The challenges with AI regarding export of porcine genetics have been discussed earlier. Although sensitive to chilling and highly susceptible to intracellular ice formation, recent progress in oocyte and embryo cryopreservation is promising (Saragusty and Arav, 2011; Cuello *et al*., 2016; Nohalez *et al*., 2018). Porcine embryos have the potential to substantially accelerate genetic gain in pig populations and to facilitate international transport of genetics, while decreasing the carbon footprint due to reduced live animal transportation. New knowledge on ET in sows is therefore essential. ET in pigs was described for the first time in 1950 at the Pig Breeding Research Institute in Poltava, Ukraine (Kvasnitski, 1950). To our knowledge, no standardized and commercial ET service in sows exist. Today, porcine ET is carried out in private companies and institutes engaged in biomedical research (Petersen *et al*., 2008; Zheng *et al*., 2016). The main oocyte source for *in vitro* maturation (IVM), *in vitro* fertilization (IVF) and *in vitro* culture (IVC) is ovaries mainly from prepuberal sows collected from the local slaughterhouse. Antral follicles are punctured in the laboratory for oocyte collection. Embryo collection after slaughter has the disadvantage of using donor sows only once. Additionally, the stage of the oestrus cycle at slaughter or the reason for slaughter is commonly unknown. Therefore, when oocytes are recovered in this manner, they are heterogenous in terms of developmental competence (Bertoldo *et al*., 2012).

To establish a viable, commercial ET concept, oocyte and embryo retrieval should be feasible for trained veterinarians under field conditions. This suggests non-surgical oocyte or embryo retrieval. Recent reports by Björkman et al. (2017b) and Yun et al. (2017) are encouraging as transvaginal ultrasound-guided biopsies of the ovaries may not cause appreciable pain or distress in non-sedated sows. However, no successful non-surgical embryo collection has been reported in pigs, except for the studies of Hazeleger et al. (1989) and Kobayashi et al. (1989) that used surgical resection of uterine horns (Brüssow *et al*., 2000). The major reason for this restriction is the anatomy of the porcine genital tract.

Non-surgical ovum pick-up (OPU) has not gained significant importance in live sows. This is probably also due to anatomical challenges and the fact that sow ovaries must be placed near the cervix for proper visualization before transvaginal follicle puncture and oocyte isolation can be conducted. Rectal palpation of pig ovaries in can be challenging due to the long uterus horns and the limited length of the rectal mesentery (Okuyama *et al*., 2017). A recent report from Japan investigated transvaginal OPU and examined the effects of different aspiration vacuum pressures and the phases of oestrous cycle on oocyte recovery, the morphology of cumulus oocyte complexes (COCs) and blastocyst formation in Berkshire pigs. The proportion of oocytes with several compact cumulus layers in 90 mmHg (27.2%) was significantly higher (P < 0.01) than in 120 mmHg (5.2%). The OPU technique enables repeated oocyte collection from highly valuable live pigs (Ikoma *et al*., 2014).

IVM, IVF and IVC have been extensively investigated in pigs taking known obstacles such as polyspermy into account (Romar *et al*., 2012; Yuan and Krisher 2012; Gil *et al*., 2017). IVM influences both nuclear and cytoplasmatic maturation of porcine oocytes and therefore pronuclear formation and cleavage (Laurincik *et al*., 1994). By modifying maturation media via addition of thiols and organic osmolytes, low incidents of male pronuclear formation after IVF can been counteracted (Funahashi and Day 1993). Polyspermic penetration of porcine oocytes range between 13% and 90% (Niwa, 1993). By simulating the oviductal environment, polyspermy is reduced and the final IVF increases the final efficiency by more than 48%. This was seemingly due to reduced sperm motility and lower capacitating status (Soriano-Úbeda *et al*., 2017). For IVC, NCSU23 containing taurine and hypotaurine promote the highest success rates in development from the single cell to blastocyst stage (Brüssow *et al*., 2000).

The selection of recipient gilts or sows will have a major impact on ET results (Brüssow *et al*., 2018). Recipient sows must be hormonally synchronous to the donors. Both the breed of the recipients and the recipient uterine environment can influence the ET results. Meishan pigs have been suggested as recipients due to their higher placental efficiency (Wilson *et al*., 1998), and post-ovulatory AI followed by ET could increase ET efficiency (Martynenko *et al*., 2004).

Embryos have mainly been transferred surgically into recipients, either into the oviducts or the cranial tip of the uterus. The need for surgical ET has certainly hampered the progress of bringing this method closer to implementation under field conditions. Multiple research groups have attempted to develop a nonsurgical ET procedure (Galvin *et al*., 1994; Hazeleger and Kemp 1994; Li *et al*., 1996; Yonemura *et al*., 1996; Martinez *et al*., 2004; Martinez *et al*., 2016).

Considerable research effort is still necessary before ET can be offered as a commercial breeding tool (Tab. 3). In conclusion, the prospects for ET in pigs have improved with recent developments in cryopreservation of oocytes and embryos. However, despite some recent developments, repeated collection of oocytes from live animals and the need for a surgical ET remain as bottlenecks for wider commercial use of ET in genetic improvement and international trade.

**Table 3:** *In vitro* and *in vivo-*related embryo transfer (ET) technologies in sows.

	Procedure	Need for research and development	References
I.	Selection of the indicated sows with superior fertility traits	Follicular fluid composition, seasonal infertility and follicle size effects on oocyte developmental competence and embryonic survival.	Peltoniemi *et al*., 1999; Bertoldo *et al*., 2013; Da Silva *et al*., 2018.
I.	Oocyte/ embryo retrieval from donor sows	Flushing equipment for sows, skill acquisition *in vivo*	Hazeleger *et al*., 1989; Kobayashi *et al*., 1989; Brüssow and Rátky 1996; Besenfelder *et al*., 1997
	Ovum pick-up (OPU) in donor sows	OPU device for sows, OPU technique optimization, skill acquisition on live animals	Brüssow *et al*., 1997; Antosik *et al*., 2007; Ikoma *et al*., 2014
II.	Gametes: *In vitro*	*In vitro* maturation (IVM), fertilization (IVF) and culture	Funahashi and Day 1993; Laurincik *et al*.,, 1994; Brüssow *et al*., 2000; Romar *et al*., 2012; Gil *et al*., 2017; Soriano-Úbeda *et al*., 2017
	Oocytes/ embryos: *In vitro*	Cryopreservation/ vitrification of embryos/ oocytes	Berthelot *et al*., 2000; Cuello *et al*., 2016; Nohalez *et al*., 2018
III.	Recipient sow synchronization	Hormonal synchronization protocol	Wilson *et al*., 1998; Martynenko *et al*., 2004; Brüssow *et al*., 2018
	ET on recipient sows	ET into the cranial portion of the corpus uteri	Webel *et al*.,, 1970; Galvin *et al*.,, 1994; Hazeleger and Kemp 1994; Li *et al*., 1996; Yonemura *et al*., 1996; Rátky *et al*., 2001; Martinez *et al*., 2004; Martinez *et al*., 2016

## Conclusions

Management of the large litters of the present hyperprolific breeds involve a sufficient appreciation of the physiological and behavioural needs of the sow prior to and around farrowing. Meeting these needs improve the capacity of the sow to produce adequate colostrum, the quantity and quality of which can be managed and monitored by modern tools such as the Brix test. Feeding sows with higher levels of fibre and decreasing the time lapse between last feeding prior to onset of parturition provide new insights for the management of parturition. Use of intermittent suckling will hasten the production cycle while extending the lactation length of small piglets. This would make the process of weaning easier for piglets, but metabolically more demanding for the sow. Recent developments in real-time ultrasonography, together with ultrasound-guided biopsy techniques provide new and novel means to study inflammatory processes of the mammary gland, dysfunction of the uterus and the ovary, timing of AI and seasonal infertility. Advancements in cryopreservation of semen, oocytes and embryos appear encouraging in terms of establishing a foundation for further development of breeding, ET and trade of germ cells and embryos across borders. While litter size in domestic European pig breeds has doubled over the past two decades, duration of farrowing has extended four to five-fold. The birth weight of piglets and colostrum intake per piglet continue to decrease. These challenges of modern breeding need to be addressed in the future. We also urge more research into this area to resolve these emerging challenges of the hyperprolific sows lines.

## References

[B001] Akdag F, Arslan S, Demir H (2009). The Effect of parity and litter size on birth weight and the effect of birth weight variations on weaning weight and pre-weaning survival in piglet. J Anim Vet Adv.

[B002] Algers B, Uvnäs-Moberg K (2007). Maternal behavior in pigs. Horm Behav.

[B003] Antosik P, Jaśkowski JM, Jeziorkowski M, Lechnowicz J (2007). The influence of vaccum pressure on quality and number of recovered oocytes aspirated from ovarian follicles of swine and cows. Arch Tierz Dummerstorf.

[B004] Baer C, Bilkei G (2005). Ultrasonographic and gross pathological findings in the mammary glands of weaned sows having suffered recidiving mastitis metritis agalactia. Reprod Domest Anim.

[B005] Beaulieu AD, Aalhus JL, Williams NH, Patience JF (2010). Impact of piglet birth weight, birth order, and litter size on subsequent growth performance, carcass quality, muscle composition, and eating quality of pork. J Anim Sci.

[B006] Berthelot F, Martinat-Botté F, Locatelli A, Perreau C, Terqui M (2000). Piglets Born after Vitrification of Embryos Using the Open Pulled Straw Method. Cryobiology.

[B007] Bertoldo MJ, Holyoake PK, Evans G, Grupen CG (2012). Seasonal variation in the ovarian function of sows. Reprod Fertil Dev.

[B008] Bertoldo MJ, Nadal-Desbarats L, Ge’rard N, Dubois A, Holyoake PK, Grupen CG (2013). Differences in the metabolomic signatures of porcine follicular fluid collected from environments associated with good and poor oocyte quality. Reproduction.

[B009] Besenfelder U, Mödl J, Müller M, Brem G (1997). Endoscopic embryo collection and embryo transfer into the oviduct and the uterus of pigs. Theriogenology.

[B010] Björkman S, Oliviero C, Hasan S, Peltoniemi O (2017). Mammary gland edema as a cause of postpartum dysgalactia in the sow-a case report. Reprod Domest Anim.

[B011] Björkman S, Yun J, Niku M, Oliviero C, Soede NM, Peltoniemi OAT (2017). Serial transvaginal ultrasound-guided biopsy of the porcine corpus luteum *in vivo.*. Reprod Fertil Dev.

[B012] Björkman S, Oliviero C, Rajala-Schultz PJ, Soede NM, Peltoniemi OAT (2017). The effect of litter size, parity, farrowing duration on placenta expulsion and retention in sows. Theriogenology.

[B013] Björkman S, Grahofer A, Han T, Oliviero C, Peltoniemi O (2018). Severe udder edema as a cause of reduced colostrum quality and milk production in sows - a case report.

[B014] Björkman S, Han T, Oliviero C, Peltoniemi O (2018). Biopsy of mammary gland in sows: A tool for studying colostrum production. Reprod Domest Anim.

[B015] Björkman S, Oliviero C, Kauffold J, Soede NM, Peltoniemi OAT (2018). Prolonged parturition and impaired placenta expulsion increase the risk of postpartum metritis and delay uterine involution in sows. Theriogenology.

[B016] Bontempo V, Sciannimanico D, Pastorelli G, Rossi R, Rosi F, Corino C (2004). Dietary conjugated linoleic acid positively affects immunologic variables in lactating sows and piglets. J Nutr.

[B017] Brüssow KP, Rátky J (1996). Endoscopic collection of porcine embryos. Reprod Dom Anim.

[B018] Brüssow KP, Torner H, Rátky J, Hunter MG, Nürnberg G (1997). Ovum pick up in swine: The influence of aspiration vacuum pressure on oocyte recovery from preovulatory follicles. Acta Vet Hung.

[B019] Brüssow KP, Torner H, Kanitz W, Rátky J (2000). *In vitro* technologies related to pig embryo transfer. Reprod Nutrit Dev.

[B020] Brüssow KP, Rátky J, Antosik P, Kempisty B, Jaśkowski JM (2018). Embryo transfer in swine - an indispensable key for the application of reproductive techniques. Electronic Journal of Polish Agricultural Universities.

[B021] Cartee RE, Powe TA, Gray BW, Hudson RS, Kuhlers DL (1986). Ultrasonographic evaluation of normal boar testicles. Am J Vet Res.

[B022] Clark SG, Althouse GC (2002). B-mode ultrasonographic examination of the accessory sex glands of boars. Theriogenology.

[B023] Clark SG, Schaeffer DJ, Althouse GC (2003). B-mode ultrasonographic evaluation of paired testicular diameter of mature boars in relation to average total sperm numbers. Theriogenology.

[B024] Chen TY, Turpin DL, Knight AL, Bouwman EG, Soede NM, Kirkwood RN, Langendijk P (2017). Lactational oestrus and reproductive performance following a delayed limited nursing schedule in primiparous sows. Theriogenology.

[B025] Corino C, Pastorelli G, Rosi F, Bontempo V, Rossi R (2009). Effect of dietary conjugated linoleic acid supplementation in sows on performance and immunoglobulin concentration in piglets. J Anim Sci.

[B026] Cuello C, Martinez CA, Nohalez A, Parrilla I, Roca J, Gil MA, Martinez EA (2016). Effective vitrification and warming of porcine embryos using a pH-stable, chemically defined medium. Sci Rep.

[B027] Curtis SE (1970). Environmental-thermoregulatory interactions and neonatal piglet survival. J Anim Sci.

[B028] Da Silva CLA, Mulder HA, Broekhuijse MLWJ, Kemp B, Soede NM, Knol EF (2018). Relationship between the estimated breeding values for litter traits at birth and ovarian and embryonic traits and their additive genetic variance in gilts at 35 days of pregnancy. Front Genet.

[B029] Decaluwé R, Maes D, Declerck I, Cools A, Wuyts B, De Smet S, Janssens G (2013). Changes in back fat thickness during late gestation predict colostrum yield in sows. Animal.

[B030] De Rensis F, Kirkwood RN (2016). Control of estrus and ovulation: Fertility to timed insemination of gilts and sows. Theriogenology.

[B031] Declerck I, Sarrazin S, Dewulf J, Maes D (2017). Sow and piglet factors determining variation of colostrum intake between and within litters. Animal.

[B032] Devillers N, Farmer C, Le Dividich J, Prunier A (2007). Variability of colostrum yield and colostrum intake in pigs. Animal.

[B033] Devillers N, Le Dividich J, Prunier A (2011). Influence of colostrum intake on piglet survival and immunity. Animal.

[B034] Difilippo E, Bettonvil M, Willems RH, Braber S, Fink-Gremmels J, Jeurink PV, Schoterman MH, Gruppen H, Schols HA (2015). Oligosaccharides in Urine, Blood, and Feces of Piglets Fed Milk Replacer Containing Galacto-oligosaccharides. J Agric Food Chem.

[B035] Donovan TS, Dritz SS (2000). Effect of split nursing on variation in pig growth from birth to weaning. J Am Vet Med Assoc.

[B036] Farmer C, Quesnel H (2009). Nutritional, hormonal, and environmental effects on colostrum in sows. J Anim Sci.

[B037] Farmer C, Maes D, Peltoniemi OAT, Zimmerman Karriker, Ramirez Schwartz, Stevenson, Zhang (2019). Mammary system.. Diseases of swine.

[B038] Feyera T, Pedersen T, Krogh U, Foldager L, Theil P (2018). Impact of sow energy status during farrowing on farrowing kinetics, frequency of stillborn piglets, and farrowing assistance. J Anim Sci.

[B039] Ford JJ, Wise TH (2011). Assessment of pubertal development of boars derived from ultrasonographic determination of testicular diameter. Theriogenology.

[B040] Gerjets I, Kemper N (2009). Coliform mastitis in sows: a review. J Swine Health and Prod.

[B041] Funahashi D, Day BN (1993). Effect of follicular fluid at fertilization *in vitro* on sperm penetration in pig oocytes. J Reprod Fertil.

[B042] Galvin JM, Killian DB, Stewart ANV (1994). A procedure for successful nonsurgical embryo transfer in swine. Theriogenology.

[B043] García-Vázquez FA, Mellagi APG, Ulguim RR, Hernandez-Caravaca I, Llamas-Lopez PJ, Bortolozzo FP (2019). Post-cervical artificial insemination in porcine: The technique that came to stay. Theriogenology.

[B044] Gerritsen G, Soede NM, Langendijk P, Hazeleger W, Kemp B (2008). The Intermittent Suckling Regimen in Pigs: Consequences for Reproductive Performance of Sows. Reprod Dom Anim.

[B045] Gil MA, Nohalez A, Martinez CA, Ake-Villanueva JR, Centurion-Castro F, Maside C, Cuello C, Roca J, Parrilla I, Martinez EA (2017). Effects of meiotic inhibitors and gonadotropins on porcine oocytes *in vitro* maturation, fertilization and development. Reprod Domest Anim.

[B046] Gu Z, Gao Y, Lin B, Zhong Z, Liu Z, Wang C, Li B (2011). Impacts of freedom farrowing pen design on sow behaviours and performance. Prev Vet Med.

[B047] Han T, Borkman S, Oliviero C, Peltoniemi O (2018). Mammary gland biopsy does not affect colostrum yield of sows: a pilot study. Reprod Domest Anim.

[B048] Hasan S, Junnikkala S, Valros A, Peltoniemi O, Oliviero C (2016). Validation of Brix refractometer to estimate colostrum immunoglobulin G content and composition in the sow. Animal.

[B049] Hasan S, Saha S, Junnikkala S, Orro T, Peltoniemi O, Oliviero C (2018). Late gestation diet supplementation of resin acid-enriched composition increases sow colostrum immunoglobulin G content, piglet colostrum intake and improve sow gut microbiota. Animal.

[B050] Hasan S, Orro T, Junnikkala S, Valros A, Peltoniemi O, Oliviero C (2019). Factors affecting sow colostrum yield and composition, and their impact on piglet growth and health. Livest Sci.

[B051] Hazeleger W, van der Meulen J, van der Lende T (1989). A method of transcervical embryo collection in the pig. Theriogenology.

[B052] Hazeleger W, Kemp B (1994). Farrowing rate and litter size after transcervical embryo transfer in sows. Reprod Domest Anim.

[B053] Herlta C, Starka R, Sigmarsson H L, Kauffold J (2018). Feasibility of transabdominal Doppler sonography for studying uterine blood flow characteristics in cycling gilts. Tierärztliche Praxis.

[B054] Herpin P, Hulin JC, Le Dividich J, Fillaut M (2001). Effect of oxygen inhalation at birth on the reduction of early postnatal mortality in pigs. J Anim Sci.

[B055] Hoy S, Lutter C, Wähner M, Puppe B (1994). The effect of birth weight on the early postnatal vitality of piglets. Dtsch Tierarztl Wochenschr.

[B056] Hurley W., Farmer C. (2015). Composition of sow colostrum and milk.. The gestating and lactating sow..

[B057] Ikoma E, Suzuki C, Ishihara Y, Komura K, Ookoda T, Maruno H (2014). Transvaginal ultrasound-guided ovum pick up (OPU) in Berkshire breed during natural estrous cycle. JPN J Swine Sci.

[B058] Islas-Fabila P, Mota-Rojas D, Martínez-Burnes J, Mora-Medina P, González-Lozano M, Roldan-Santiago P (2018). Physiological and metabolic responses in newborn piglets associated with the birth order. Anim Reprod Sci.

[B059] Jensen P. (1986). Observations of the maternal behaviour of free-ranging domestic pigs. Appl Anim Behav Sci.

[B060] Kaiser M, Jacobsen S, Haubro-Andersen P, Bækbo P, Cerón J, Dahl J, Escribano D, Jacobson M (2018). Inflammatory markers before and after farrowing in healthy sows and in sows affected with postpartum dysgalactia syndrome. BMC Vet Res.

[B061] Kaiser M, Jacobsen S, Haubro-Andersen P, Bækbo P, Cerón J, Dahl J, Escribano D, Theil P, Jacobson M (2018). Hormonal and metabolic indicators before and after farrowing in sows affected with postpartum dysgalactia syndrome. BMC Vet Res.

[B062] Kauffold J, Althouse GC (2007). An update on the use of B-mode ultrasonography in female pig reproduction. Theriogenology.

[B063] Kauffold J, Kessler M, Richter A, Beynon N, Wehrend A (2011). B‐mode Ultrasound and Grey‐Scale Analysis of the Epididymis in Boars, and the Relationship to Semen Parameters. Reprod Domest Anim.

[B064] Kauffold J, Wehrend A (2014). Fertilitätsstörungen beim weiblichen Schwein. Tierärztliche Praxis Ausgabe G: Großtiere/Nutztiere.

[B065] Kemp B, Soede N (2012). Reproductive Issues in Welfare-Friendly Housing Systems in Pig Husbandry: A review. Reprod Domest Anim.

[B066] Kielland C, Rootwelt V, Reksen O, Framstad T (2015). The association between immunoglobulin G in sow colostrum and piglet plasma. J Anim Sci.

[B067] Kirkden RD, Broom DM, Andersen IL (2013). Invited review: piglet mortality: management solutions. J Anim Sci.

[B068] Klobasa F, Butler JE (1987). Absolute and relative concentrations of immunoglobulins G, M, and A, and albumin in the lacteal secretion of sows of different lactation numbers. Am J Vet Res.

[B069] Kobayashi K, Hayashi S, Ohtubo Y, Honda A, Mizuno J, Hirano S. (1989). Theriogenology.

[B070] Kvasnitski AV (1950). The research on interbreed ova transfer in pigs. Social Livest Breed J.

[B071] Langendijk P. (2015). Early gestation feeding and management for optimal reproductive performance.. The gestating and lactating sow..

[B072] Laurincik J, Rath D, Niemann H (1994). Differences in pronucleus formation and first cleavage following *in vitro* fertilization between pig oocytes matured *in vivo* and *in vitro .*. J Reprod Fertil.

[B073] Li J, Rieke A, Day BN, Prather RS (1996). Technical note: porcine non-surgical embryo transfer. J Anim Sci.

[B074] Manjarin R, Montano YA, Kirkwood RN, Bennet DC, Petrovski KR (2018). Effect of piglet separation from dam at birth on colostrum uptake. Can J Vet Res.

[B075] Martineau GP, Farmer C, Peltoniemi OAT, Zimmerman JJ, Locke A, Karriker AR, Ramirez A, Schwartz KJ, Stevenson GW (2012). Mammary system.. Diseases of swine.

[B076] Martinez EA, Vazquez JM, Roca J, Lucas X, Gil MA, Parrilla I, Vazquez JL, Day BN (2002). Minimum number of spermatozoa required for normal fertility after deep intra- uterine insemination in non-sedated sows. Reproduction.

[B077] Martinez EA, Caamano JN, Gil MA, Rieke A, McCauley TC, Cantley TC, Vazquez JM, Roca J, Vazquez JL, Didion BA, Murphy CN, Prather RS, Day BN (2004). Successful nonsurgical deep uterine embryo transfer in pigs. Theriogenology.

[B078] Martinez EA, Vazquez JM, Parrilla I, Cuello C, Gil MA, Rodriguez-Martinez H, Roca J, Vazquez JL (2006). Incidence of unilateral fertilizations after low dose deep intrauterine insemination in spontaneously ovulating sows under field conditions. Reprod Domest Anim.

[B079] Martinez EA, Nohalez A, Martinez CA, Parrilla I, Vila J, Colina I, Diaz M, Reixach J, Vazquez JL, Roca J, Cuello C, Gil MA (2016). The Recipients' Parity Does Not Influence Their Reproductive Performance Following Non-Surgical Deep Uterine Porcine Embryo Transfer. Reprod Domest Anim.

[B080] Martynenko NA, Tchjrkov AG, Denisjuk PV, Korennoij SN (2004). Transcervical transplantation of porcine embryos and neutralization of superinduction using inseminated recipients. Vistnik Poltavskoij Dershavnoij Agrarnoij Akademii.

[B081] Matheson SM, Walling GA, Edwards SA (2018). Genetic selection against intrauterine growth retardation in piglets:a problem at the piglet level with a solution at the sow level. Genetics Selection Evolution.

[B082] van Nieuwamerongen SE, Bolhuis JE, van der Peet-Schwering CMC, Soede NM (2014). A review of sow and piglet behaviour and performance in group housing systems for lactating sows. Animal.

[B083] Niwa K. (1993). Effectiveness of *in vitro* maturation and *in vitro* fertilization techniques in pigs. J Reprod Fertil Suppl.

[B084] Nohalez A, Martinez CA, Parrilla I, Roca J, Gil MA, Rodriguez-Martinez H, Martinez EA, Cuello C (2018). Exogenous ascorbic acid enhances vitrification survival of porcine *in vitro* -developed blastocysts but fails to improve the *in vitro* embryo production outcomes. Theriogenology.

[B085] Okuyama MW, Sugiura T, Moriyoshi M, Yamashita K, Tamura J, Katagiri S (2017). A transvaginal endoscopy-based technique for performing ovarian examinations in sows. J Reprod Dev.

[B086] Oliviero C, Heinonen M, Valros A, Halli O, Peltoniemi OAT (2008). Effect of the environment on the physiology of the sow during late pregnancy, farrowing and early lactation. Anim Reprod Sci.

[B087] Oliviero C, Heinonen M, Valros A, Peltoniemi OAT (2010). Environmental and sow-related factors affecting the duration of farrowing. Anim Reprod Sci.

[B088] Oliviero C., Rodriqez-Martinez H, Soede N, Flowers W (2013). Management to improve neonate piglet survival.. Control of pig reproduction IX..

[B089] Oliviero C, Kothe S, Heinonen M, Valros A, Peltoniemi O (2013). Prolonged duration of farrowing is associated with subsequent decreased fertility in sows. Theriogenology.

[B090] Oliviero C, Junnikkala S, Peltoniemi OAT (2019). The challenge of large litters on the immune system of the sow and the piglets. Reprod Domest Anim.

[B091] Peltoniemi OAT, Love RJ, Heinonen M, Tuovinen V, Saloniemi H (1999). Seasonal and management effects on fertility of the sow:a descriptive study. Anim Reprod Sci.

[B092] Peltoniemi OAT, Alm K, Andersson M (2009). Uterine insemination with a standard AI dose in a sow pool system. Reprod Domest Anim.

[B093] Peltoniemi OAT, Björkman S, Oliviero C (2016). Parturition effects on reproductive health in the gilt and sow. Reprod Domest Anim.

[B094] Peltoniemi O, Kemp B, Noakes DE, Parkinson TJ, England GCW (2019). Infertility in the pig and the control of pig herd fertility.. Veterinary Reproduction and Obstetrics.

[B095] Petersen B, Lucas-Hahn A, Oropeza M, Hornen N, Lemme E, Hassel P, Queisser AL, Niemann H (2008). Development and Evaluation of a Highly Efficient Protocol of Porcine Somatic Cloning Using Preovulatory Embryo Transfer in Peripubertal Gilts. Cloning and Stem Cells.

[B096] Pinho RO, Camilo B S, Lima DMA, Villadiego F A C, Vergara JCM, Shiomi HH, Guimarães JD (2018). The use of ultrasonography in the reproductive evaluation of boars. Reprod Domest Anim.

[B097] Porter P. (1969). Transfer of immunoglobulins IgG, IgA and IgM to lacteal secretions in the parturient sow and their absorption by the neonatal piglet. Biochim Biophys Acta.

[B098] Quesnel H, Brossard L, Valancogne A, Quiniou N (2008). Influence of some sow characteristics on within-litter variation of piglet birth weight. Animal.

[B099] Quesnel H. (2011). Colostrum production by sows:Variability of colostrum yield and immunoglobulin G concentrations. Animal.

[B100] Quesnel H, Farmer C, Theil PK, Farmer C (2015). Colostrum and milk production.. The Gestating and lactating sow..

[B101] Quigley JD, Lago A, Chapman C, Erickson P, Polo J (2013). Evaluation of the Brix refractometer to estimate immunoglobulin G concentration in bovine colostrum. J Dairy Sci.

[B102] Rátky J, Brüssow KP, Solti L, Torner H, Sarlós P (2001). Ovarian response, embryo recovery and results of embryo transfer in a Hugarian native pig breed. Theriogenology.

[B103] Roca J, Parrilla I, Bolarin A, Martinez EA, Rodriguez-Martinez H (2016). Will AI in pigs become more efficient?. Theriogenology.

[B104] Romar R, Coy P, Rath D (2012). Maturation conditions and boar affect timing of cortical reaction in porcine oocytes. Theriogenology.

[B105] Rooke JA, Bland IM (2002). The acquisition of passive immunity in the new-born piglet. Livest Prod Sci.

[B106] Rua MAS, Quirino CR, Ribeiro RB, Carvalho ECQ, Bernadino MLA, Bartholazzi Junior A, Cipagalta LF, Barreto MAP (2018). Diagnostic methods to detect uterus illnesses in mares. Theriogenology.

[B107] Saragusty J, Arav A (2011). Current progress in oocyte and embryo cryopreservation by slow freezing and vitrification. Reproduction.

[B108] Schmitt O, Baxter EM, Boyle LA, O'Driscoll K (2019). Nurse sow strategies in the domestic pig: I. Consequences for selected measures of sow welfare. Animal.

[B109] Schmitt O, Baxter EM, Boyle LA, O'Driscoll K (2019). Nurse sow strategies in the domestic pig:II. Consequences for piglet growth, suckling behaviour and sow nursing behaviour. Animal.

[B110] Senger PL (2012). The organization & function of the female reproductive tract.. Pathways to pregnancy & parturition.

[B111] Soede NM, Langendijk P, Kemp B (2011). Reproductive cycles in pigs. Animal Rep Sci.

[B112] Soede NM, Laurenssen B, Abrahamse-Berkeveld M, Gerritsen R, Dirx-Kuijkend N, Langendijk P, Kemp B (2012). Timing of lactational oestrus in intermittent suckling regimes:Consequences for sow fertility. Anim Reprod Sci.

[B113] Soede NM, Kemp B (2015). Best practices in the lactating and weaned sow to optimize reproductive physiology and performance.. The gestating and lactating sow..

[B114] Soriano-Úbeda C, García-Vázquez FA, Aguirregomezcorta R, Matás C (2017). Improving porcine in vitro fertilization output by simulating the oviductal environment. Nature Scientific Reports.

[B115] Spiegel F, Spiegel S, von Altrock A, Verspohl J, Seehusen F, Wendt M (2017). Occurrence of bacteria in milk samples and biopsy samples of the mammary gland from health sows..

[B116] Smit M, Spencer J, Almeida F, Patterson J, Chiarini-Garcia H, Dyck M, Foxcroft G (2013). Consequences of a low litter birth weight phenotype for postnatal lean growth performance and neonatal testicular morphology in the pig. Animal.

[B117] Stark R, Herlt C, Sigmarsson HL, Kauffold J (2019). Feasibility of transabdominal Doppler ultrasonography for studying ovarian blood flow characteristics in cycling gilts. Tierarztl Prax Ausg G Grosstiere Nutztiere.

[B118] Sumransap P, Tummaruk P, Kunavongkrit A (2007). Sperm distribution in the reproductive tract of sows after intrauterine insemination. Reprod Domest Anim.

[B119] Tummaruk P, Sumransap P, Techakumphu M, Kunavongkrit A (2007). Distribution of spermatozoa and embryos in the female reproductive tract after unilateral deep intra uterine insemination in the pig. Reprod Domest Anim.

[B120] Tummaruk P, Sang-Gassanee K (2013). Effect of farrowing duration, parity number and the type of anti-inflammatory drug on postparturient disorders in sows:a clinical study. Trop Anim Health Prod.

[B121] Vallet J, Miles J, Rempel L (2013). A simple novel measure of passive transfer of maternal immunoglobulin is predictive of preweaning mortality in piglets. Vet J.

[B122] Watson PF, Behan JR (2002). Intrauterine insemination of sows with reduced sperm numbers:results of a commercially based field trial. Theriogenology.

[B123] Webel SK, Peters JB, Anderson LL (1970). Synchronous and asynchronous transfer of embryos in the pig. J Anim Sci.

[B124] Wilson ME, Biensen NJ, Youngs CR, Ford SP (1998). Development of Meishan and Yorkshire littermate conceptuses in either a Meishan or Yorkshire uterine environment to day 90 of gestation and to term. Biol Reprod.

[B125] Yao W, Li J, jun Wang J, Zhou W, Wang Q, Zhu R, Wang F, Thacker P (2012). Effects of dietary ratio of n-6 to n-3 polyunsaturated fatty acids on immunoglobulins, cytokines, fatty acid composition, and performance of lactating sows and suckling piglets. J Anim Sci Biotechn.

[B126] Yeste M, Rodríguez-Gil JE, Bonet S (2016). Artificial insemination with frozen-thawed boar sperm. Mol Reprod Dev.

[B127] Yonemura I, Fujino Y, Irie S, Miura Yl (1996). Transcervical transfer of porcine embryos under practical conditions. J Reprod Dev.

[B128] Yuan Y, Krisher RL (2012). *In vitro* maturation (IVM) of porcine oocytes. Methods Mol Biol.

[B129] Yun J, Swan KM, Vienola K, Kim YY, Oliviero C, Peltoniemi OAT, Valros A (2014). Farrowing environment has an impact on sow metabolic status and piglet colostrum intake in early lactation. Livest Sci.

[B130] Yun J, Björkman S, Pöytäkangas M, Peltoniemi OAT (2017). The effects of ovarian biopsy and blood sampling methods on salivary cortisol and behaviour in sows. Res Vet Sci.

[B131] Zheng C, Jian-Tao Z, Hai-Feng L, Hong-Bin W (2016). Laparoscopic Embryo Transfer in Pigs: Knowledge for Surgical Procedures. J Northeast Agric Univ.

